# Body-piercing associated appendicitis in the pregnant patient incidentally discovered during Caesarian section

**DOI:** 10.1093/jscr/rjae021

**Published:** 2024-01-24

**Authors:** Jennifer Schadt, Andrew Hungerford, Molly Houser, Nia Zalamea

**Affiliations:** Department of Surgery, University of Tennessee Health Science Center, 910 Madison Ave, Ste 327, Memphis, TN 38163, United States; College of Medicine, University of Tennessee Health Science Center, 910 Madison Ave, Memphis, TN, 38163, United States; Department of Obstetrics and Gynecology, University of Tennessee Health Science Center, 853 Jefferson Avenue, Memphis, TN, United States; Department of Surgery, University of Tennessee Health Science Center, 910 Madison Ave, Ste 327, Memphis, TN 38163, United States; Global Surgery Institute, University of Tennessee Health Science Center, 910 Madison Avenue Suite 327, Memphis, TN, 38163, United States; Center for Multicultural and Global Health, University of Tennessee Health Science Center, 910 Madison Avenue, Memphis, TN, 38163, United States

**Keywords:** appendicitis, pregnancy, foreign body appendicitis

## Abstract

Appendicitis is the most common general surgery condition encountered in pregnant patients. Its presentation and diagnosis can be confounded by physiological changes of pregnancy causing atypical symptoms and overlap between pregnancy symptoms and appendicitis. Diagnosis of appendicitis in pregnancy is important, as pregnant persons have a much higher rate of complication from appendicitis. In this case report, we describe a patient with a history of right lower quadrant pain thought to be related to round ligament pain. Her previously undiagnosed appendicitis was discovered during routine inspection during caesarian section. The patient’s final pathology revealed a metallic foreign body, one of the rarest causes of appendicitis.

## Introduction

Appendicitis is one of the most common general surgery complaints, and its incidence peaks in the second and third decades of life, coinciding with peak childbearing years [1]. It should be no surprise that appendicitis is the most common general surgery related condition in pregnant persons, occurring in ~1 in 1500 pregnancies [1]. Not-so-common however is appendicitis due to foreign body ingestion, with a prevalence of ~0.0005% [2]. Here we report a case of undiagnosed appendicitis in pregnancy due to foreign body ingestion.

## Case report

Our patient, KC, is a 32-year-old female, G3P1102, with no significant past medical history, and a surgical history of 2 prior C-sections. She began experiencing abdominal pain, nausea, and vomiting, which she attributed to enteritis, as her young son had started experiencing similar symptoms the week prior. While her son’s symptoms resolved after a few days, her symptoms persisted, and her abdominal pain localized to the right lower quadrant.

She took a home pregnancy test, which resulted as positive. She presented to her OBGYN for persistent vomiting. She was diagnosed with hyperemesis gravidarum and sent to a high-risk maternal fetal medicine specialist who followed her throughout her pregnancy. Her nausea, vomiting, and abdominal pain subsided after a few weeks. Her fetus was diagnosed with Dandy–Walker syndrome based on routine prenatal ultrasound findings. She otherwise had an unremarkable pregnancy and was admitted at 39 weeks for a planned C-section.

The C-section was uneventful, with the delivery of a healthy infant. After infant delivery, her OBGYN surgeon noted that her appendix appeared enlarged and was adhered to the abdominal wall. General surgery was consulted intraoperatively. Visual inspection revealed a large appendix with significant adhesions between the appendiceal tip and right pelvic sidewall. Consent was obtained from the patient to perform an appendectomy at this time.

The general surgery team joined intraoperatively and proceeded with an open appendectomy. Upon closer visualization, there was appendiceal dilation from the tip of the appendix extending to 1 cm past the base with an inflamed appendiceal mesentery. There was no palpable appendicolith, though the appendix was thickened, and no purulence was noted. The adhesions between the appendix and the pelvic sidewall were taken down with electrocautery. The base of the appendix was dissected free of the cecum and the terminal ileum was visualized. The mesentery of the appendix was hemostatically ligated with 0 vicryl suture. The base of the appendix was stapled with TA 60 stapler blue. The appendix was then removed. The appendiceal stump was then imbricated and buried with lembert sutures. The remainder of the procedure was then continued by the primary team without complication.

**Figure 1:**
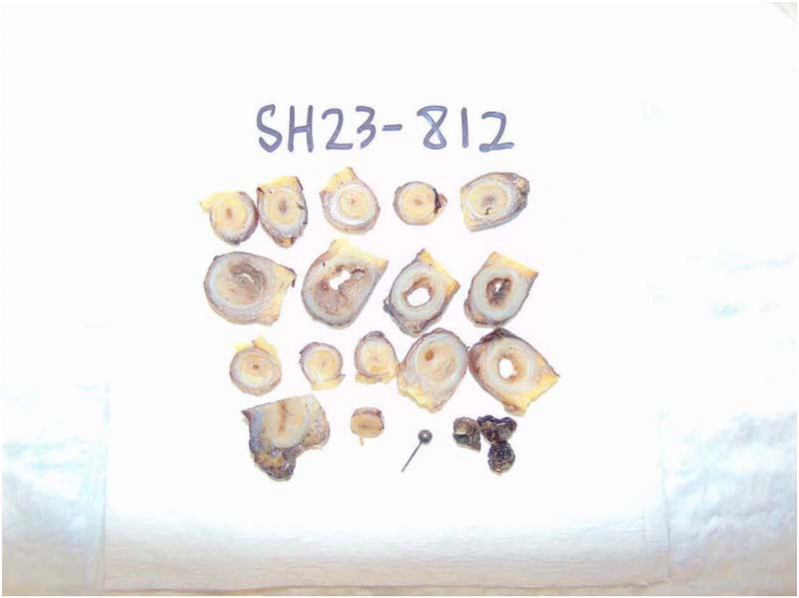
Gross pathology specimen showing transverse sections of appendix.

**Figure 2:**
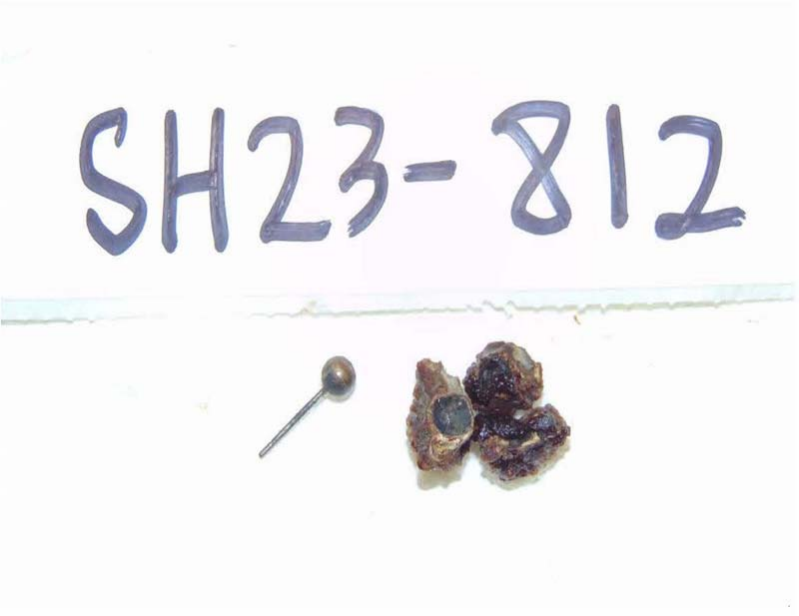
Gross pathology specimen showing fecalith and foreign body from lumen of appendix.

The patient did well postoperatively, noted the disappearance of her right lower quadrant pain, and she had a minor postoperative ileus that resolved by postoperative Day 4. Her pathology revealed a benign appendix with acute appendicitis, and periappendicitis changes to the mesentery. Within the lumen of the appendix was found a fecalith associated with a metallic foreign body, consistent with a body piercing as shown in Figs 1 and 2. The patient denied a history of any type of oral piercing. She recalled an event prior to the onset of her abdominal pain of food feeling stuck in her throat while eating at a restaurant; however it is unclear if this was the source of ingestion.

## Discussion

While the incidence of appendicitis in pregnant and non-pregnant women is approximately the same, the rate of perforated appendicitis and appendicitis-related complications are much higher in pregnancy [1]. Appendicitis and pregnancy have many overlapping symptoms, making the diagnosis of appendicitis in pregnancy difficult. The primary symptoms of appendicitis—abdominal pain, nausea, vomiting, anorexia—are quite prevalent in pregnancy. Even the classic pattern of umbilical abdominal pain eventually localizing to the right lower quadrant can be misleading in the diagnosis of appendicitis in pregnancy as the gravid uterus can displace the appendix [3]. It is important to have a high index of suspicion for appendicitis in the pregnant patient, as it can lead to devastating effects if untreated: if appendicitis progresses to abscess or peritonitis, the rate of fetal loss may be as high as 6% [1]. With free perforation, risk of fetal loss increases to 36% [1, 4]. Aside from unclear patient presentation, the diagnosis of appendicitis in the pregnant patient can be made more difficult by the dangers posed by CT scan to the developing fetus. CT is the gold standard for imaging of appendicitis, with a sensitivity approaching 100%, however it carries a risk of ionizing radiation to the fetus [5]. Ultrasound has a sensitivity of only 68.4%, and can be highly dependent upon technician experience as well as positioning of the appendix [5]. MRI shows a sensitivity in the range of 90%–100% and a specificity of ~97% [6, 7]. In the pregnant patient with abdominal pain, nausea, vomiting, and anorexia, it is appropriate to begin with ultrasound as a first-line imaging study. An ultrasound that is negative for appendicitis does not however definitively rule out the condition, and an MRI should be obtained.

The most common cause of appendicitis is a fecalith obstructing the appendiceal lumen, leading to congestion and inflammation, and eventually perforation and infection if not surgically removed or contained by the body. A foreign body, if small enough to become lodged in the appendiceal orifice, may act similarly to an obstructing fecalith. The lumen of the appendix is narrow, and once a foreign body enters the appendiceal lumen, peristalsis is insufficient to propel it into the cecum [2]. Foreign body induced appendicitis is incredibly rare, and the prevalence is ~0.0005% [2]. It is therefore remarkable that our patient not only ingested a piercing that was not her own, but that it also caused her to develop appendicitis. It is further remarkable that she did not develop complications from her appendicitis, and that it did not significantly impact her pregnancy. In this patient course, the vigilant routine inspection of the appendix by her high-risk obstetrician enabled swift recognition and treatment.
